# Recyclable dye-sensitized TiO_2_ composite membranes with interfacial spectral complementarity for sustainable white-light-driven dye degradation

**DOI:** 10.1039/d5ra09829g

**Published:** 2026-02-05

**Authors:** Hongyang Cen, Wei Zhu, Yongqiang Li, Yajing Song, Zhenxin Xu, Pengjiang Tan, Shuo Cao, Yonglei Gao, Yi Huang

**Affiliations:** a State Key Laboratory of Bio-based Fiber Materials, Zhejiang Sci-Tech University Hangzhou 310018 China yqqli@163.com; b Engineering Research Center for Eco-Dyeing and Finishing of Textiles, Ministry of Education, Zhejiang Sci-Tech University Hangzhou 310018 China; c Key Laboratory of Intelligent Textile and Flexible Interconnection of Zhejiang Province Hangzhou 310018 China; d Tongxiang Research Institute, Zhejiang Sci-Tech University Tongxiang 314500 China

## Abstract

The efficient and low-energy treatment of dye wastewater remains a significant challenge. Herein, a novel co-sensitized TiO_2_ photocatalyst (CS-TiO_2_) was constructed by combining ruthenium-based dye N719 with a laboratory-synthesized organic dye RA, aiming to extend the visible-light absorption range. The CS-TiO_2_ was subsequently embedded into poly(methyl methacrylate) micro–nano fibers *via* centrifugal spinning, yielding easily recyclable photocatalytic membranes. After deducting the 30% self-degradation contribution of methylene blue arising from its intrinsic photosensitizing effect, the as-prepared PMMA/CS-TiO_2_ membrane achieved a net MB degradation efficiency of 58.12%—significantly superior to that of single-dye sensitized counterparts. This enhanced performance is ascribed to efficient charge separation and boosted production of dominant ·OH radicals enabled by the synergistic co-sensitization effect. Notably, the membrane retained ∼80% of its initial net degradation efficiency after five consecutive cycles, demonstrating excellent reusability and structural stability. This work offers a promising approach for constructing efficient, sustainable, and recyclable photocatalytic systems for dye wastewater remediation.

## Abstract 2

The efficient and low-energy treatment of dye wastewater remains a significant challenge.

## Graphical abstract

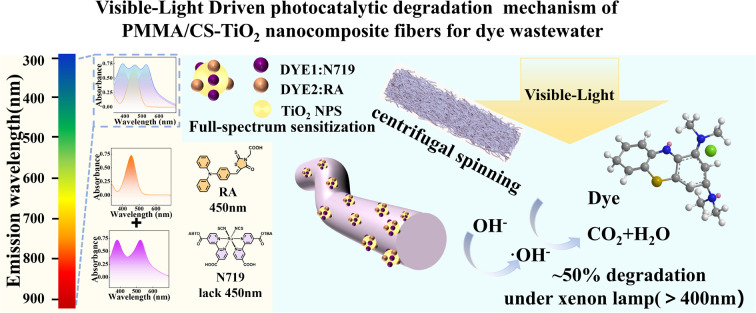

## 1. Introduction

With the advancement of science and the rapid development of the contemporary chemical industry, environmental pollution problems have become increasingly severe, among which water pollution is particularly serious.^1–3^ The textile industry, as a traditional manufacturing sector, is one of the industries with high labor intensity, large water consumption, and serious pollution.^4^ As the world's largest exporter of textiles, China's annual water consumption and wastewater discharge are astonishing. The composition of this wastewater is extremely complex, often containing acids, bases, dyes, hydrogen peroxide, starch, surfactants, dispersants, and other chemical substances, and usually has a strong color and high concentration of organic compounds.^5–7^ Advanced oxidation processes (AOPs) stand out in this environment, relying on the *in situ* generation of powerful oxidants, especially hydroxyl radicals, to mineralize organic pollutants into harmless end products such as CO_2_ and H_2_O.^8–10^ Compared to traditional sewage treatment methods, AOPs can transfer pollutants or produce harmful by-products at different stages, achieving complete degradation without generating secondary pollution. Among them, photocatalysis is a widely studied AOP, which utilizes light energy in the presence of semiconductors to activate redox reactions.^11^ Since the scientist Fujishima first discovered the photocatalytic properties of TiO_2_ in 1972,^12^ the research popularity of TiO_2_ has been increasing year by year due to its readily available raw materials, high stability, and low biological toxicity.^13,14^ The bandgap of TiO_2_ is approximately 3.2 eV. The wide bandgap characteristic means that it can only absorb ultraviolet light with a wavelength of less than 380 nm. Meanwhile, the electron–hole recombination rate of TiO_2_ is relatively high, which significantly reduces the photocatalytic efficiency of TiO_2_.^15^ To overcome this shortcoming, dye sensitization strategies are widely adopted. By introducing organic or metal complex dyes as photosensitizers, the light response range can be extended to the visible light region.^16^ The core of photocatalysis lies in the semiconductor catalyst itself, absorbing photons to generate electron–hole pairs, which initiate redox reactions through charge separation. The catalyst is not consumed in the reaction but merely serves as the “core driving unit” for light response.^17^ Photosensitization, on the other hand, relies on photosensitizers as “light-harvesting antennas”. After absorbing photons and being excited, they inject high-energy electrons into the conduction band of the semiconductor to expand the light response range, while the photosensitizers themselves are oxidized and require regeneration through the electron donor in the system.^18^ In dye-sensitized TiO_2_ systems, pure photosensitization can only achieve electron injection, but combining it with the charge separation characteristics of photocatalysis can further optimize carrier utilization efficiency. Nevertheless, a single type of dye sensitization system struggles to simultaneously meet the three core requirements of wide spectral response, efficient charge transfer, and environmental compatibility. This has become a key bottleneck restricting the practical application of dye-sensitized TiO_2_ photocatalytic technology.^19^ Therefore, developing a multi-dye co-sensitization strategy, integrating the structural and performance advantages of different dyes to achieve complementary benefits, has become an important research direction to overcome the aforementioned limitations.

Previous studies have reported various visible light active photocatalysts for pollutant degradation, including precious metal modified TiO_2_, transition metal doped TiO_2_, and naturally dye-sensitized TiO_2_ systems.^20^ For example, Pd/TiO_2_ nanocomposites synthesized by microplasma achieved 96.8% degradation of Rhodamine B within 15 minutes under xenon lamp irradiation,^21^ while copper-doped three-phase TiO_2_ (rutile perovskite) achieved 86.1% degradation of methylene blue under 66 W LED illumination.^22^ However, there are problems such as expensive metal nanoparticles and long-term stability damage caused by dopant leaching. TiO_2_ sensitized with natural dyes has ecological compatibility under visible light LED lamps, with a methylene blue degradation efficiency of 60%, but its activity significantly decreases after multiple cycles.^23^ In contrast, our work focuses on the stepwise co-sensitization of titanium dioxide with ruthenium-based dye N719 and aggregation-induced luminescent active organic dye RA. This system expands the visible light absorption range and suppresses dye aggregation and photobleaching through synergistic energy level matching.^24^ Importantly, integrating the composite material into the fiber membrane structure not only achieves high degradation efficiency of methylene blue, but also convenient recyclability through simple flushing and filtration, and maintains initial activity after five cycles—overcoming the tedious separation problem of powder catalysts. This study systematically compared the photocatalytic performance, photoresponsive range, and long-term reusability, aiming to position co-sensitized titanium dioxide fiber membranes as an efficient, sustainable, and practical balanced solution for visible light-driven wastewater treatment.

## 2. Experimental

### 2.1. Materials and instruments

Polyethylene terephthalate (PMMA, Mw = 1 00 000–1 20,000), *N*,*N*-dimethylformamide (DMF), nano-titanium dioxide (TiO_2_ NPS) P25 20 nm, bis(tetrabutylammonium), dihydrobis (isothiocyanate), bis(2,2′-dipyridine-4,4′-diformic acid), ruthenium(ii) (N719), diphenylamino-4-benzaldehyde, 3-carboxymethyl tannin, ammonium acetate, acetic acid, ethanol. The above drugs were purchased from Shanghai Maclin Biochemical Technology Co., LTD. Methylene blue (MB) and acid red 3 59 200% was purchased from Zhejiang Hengsheng Dyeing and Finishing Co., LTD. (Zhejiang, China). All chemicals are of analytical grade and can be used directly without further purification. RA was synthesized in the laboratory (Fig. S1–S3).

Lab-made deionized water was prepared in an Ultra-pure water meter (D24 UV, Merck Milliporesh, Germany).

### 2.2. Methods

#### 2.2.1. Preparation of dye-sensitized titanium dioxide photocatalyst

Dye solutions were prepared by dissolving individual N719 and RA sensitizers in ethanol (denoted as N719-TiO_2_ and RA-TiO_2_). Using a stepwise sensitization approach, TiO_2_ nanoparticles were first immersed in the RA solution for 2 h, then centrifuged, rinsed, and subsequently soaked in the N719 solution for 12 h. After further rinsing, centrifugation, and drying, the co-sensitized TiO_2_ (denoted as CS-TiO_2_) was obtained.^25^

#### 2.2.2. Centrifugal spinning preparation of dye-sensitized titanium dioxide composite fibers

**Fig. 1:**
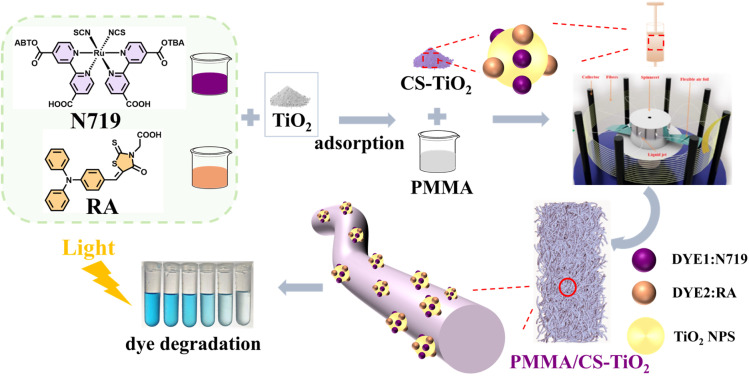
Schematic illustration of dye-sensitized TiO_2_ composite micro–nano fibers with photocatalytic degradation of dye wastewater.

As shown in Fig. 1, the spinning solution was prepared by dissolving TiO_2_ and PMMA in *N*,*N*-dimethylformamide (DMF) under constant-temperature stirring for 24 h. Subsequently, the solution was loaded into a syringe and centrifugally spun through a 0.4 mm nozzle at 12 000 rpm, with fibers collected on a rod 12 cm away.

### 2.3. Adsorption isotherm

Batch adsorption analysis was conducted in a sample flask (50 mL). The conical flask contained 10 mL of RA dye ethanol solution and N719 dye ethanol solution with concentrations of 5 × 10^−6^, 1 × 10^−5^, 2 × 10^−5^, 5 × 10^−5^, and 1 × 10^−4^ mmol L^−1^ 0.01 g of nano-TiO_2_ was added, followed by ultrasonic treatment for 30 minutes. And stir at 500 rpm for 5 hours using a stirrer. The residual concentrations of RA dye and N719 dye in ethanol solution were determined by spectrophotometry. The amount of dye adsorbed onto the nano-TiO_2_ adsorbent (*i.e.*, equilibrium adsorption) was determined by the following equation:

1 *q*_e_ = (*C*_0_ − *C*_e_)*V*/*m*

Among them, *q*_e_ (mmol g^−1^) represents the dye equilibrium adsorption capacity per unit mass of the adsorbent, *C*_0_ (mmol L^−1^) is the initial dye concentration, *C*_e_ (mmol L^−1^) is the equilibrium dye concentration, *V* (L) is the volume of the dye solution, and *m* (g) is the dry weight of nano-TiO_2_.

### 2.4. Photocatalytic experiments

The photocatalytic activity of the prepared PMMA/TiO_2_-composite fiber was evaluated by a photochemical reactor (Xujiang Electromechanical Plant, Nanjing, China) to degrade MB and acid red 3 59 200% dye solution. The experimental conditions are as follows: the temperature was maintained at 25 °C (regulated by circulating water), the xenon lamp was operated at a power of 500 W equipped with a filter to cut off UV radiation with wavelengths shorter than 400 nm. The light intensity irradiated on the sample surface was calibrated as 52.5 mW cm^−2^ using a calibrated optical power meter, and the stirring speed was set at 600 rpm. The details of the experiment are as follows: 0.1 g composite fiber is immersed in the prepared dyeing solution, 50 mL dyeing solution is taken in quantity, the dyeing solution is treated in dark condition for 3 h, the fiber reaches the adsorption equilibrium, and the 50 mL degradation target is re-quantified for photocatalytic reaction for 1 h. Seven groups of samples were sampled continuously at 4 mL every 10 min. The same sample was reused five times. The absorbance value of each sample was tested using a UV spectrophotometer. The degradation rate *η* was calculated from formula (2).^26,27^

2 *η* = (*C*_0_ − *C*_*t*_)/*C*_0_ × 100%

where *C*_0_ represents the initial concentration of dye solution, *C*_*t*_ the concentration of dye solution after *t* degradation, and *η* the dye decolorization rate.

### 2.5. Theoretical calculations

The energy-minimum configuration and electrostatic potential (ESP) maps of RA were obtained using the B3LYP method of densityfunctional theory (DFT) based on the 6-31G(d) (for C, H, O, N, S) basis sets on Gaussian 09 W software.^28,29^ Time-Dependent DFT (TD-DFT) calculations were performed to determine the frontier molecular orbital distributions (HOMO and LUMO) and electronic transitions.

### 2.6. Characterization

High-resolution mass spectrometry (HRMS) was performed on a Waters ESI mass spectrometer. NMR spectra were recorded on a Waters G2-S Tof mass spectrometer. The surface morphology of the composite fibers was examined using a field-emission scanning electron microscope (GemiSEM 500, Zeiss, Germany). The surface composition was analyzed by Fourier transform infrared spectroscopy (FTIR) on a Nicolet 5700 spectrometer (ATR mode, Thermo Fisher, USA). The surface elemental composition was quantitatively determined by X-ray photoelectron spectroscopy (XPS) on a Thermo Fisher K-Alpha spectrometer. The mechanical properties of the modified fabrics were tested with an electronic fabric strength tester (YG(B)026 G, Wenzhou Darong Textile Instrument Co., Ltd). The optical properties of the fibers were analyzed using a fluorescence spectrophotometer (F-4600, Hitachi, Japan), and the visible absorption spectra of the dyes were measured by a UV-vis spectrophotometer (UV-2600, Shimadzu, Japan). Free radicals were detected using an EPR 200-Plus X-band electron paramagnetic resonance (EPR) spectrometer.

## 3. Results and discussion

### 3.1. Characterization of RA

**Fig. 2:**
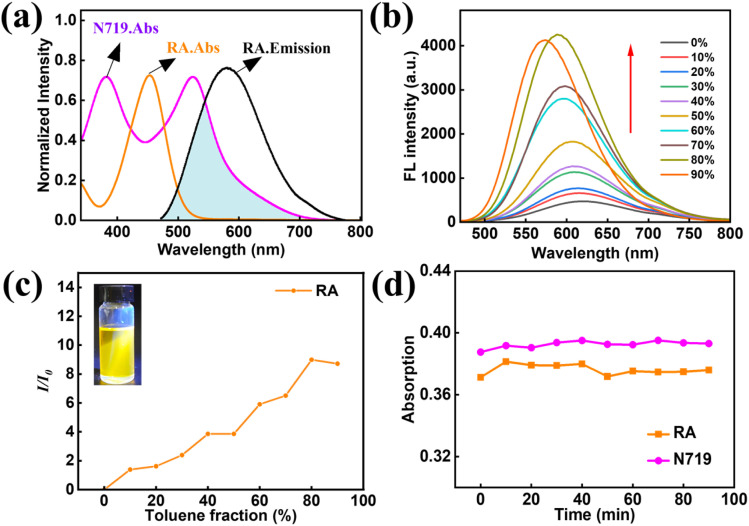
(a) Normalized absorption and fluorescence emission spectra of RA and N719 in ethanol solution; (b) fluorescence spectra of RA in different toluene components (DMSO/toluene mixed solvent); (c) the relative fluorescence spectra of RA in different toluene components (DMSO/toluene mixed solvent); (d) absorbance changes of RA and N719 ethanol solutions under 500 W xenon lamp irradiation (measured every 10 min).

To demonstrate the FRET process between RA and N719, the UV-vis absorption and fluorescence emission spectra of RA and N719 in ethanol were first analyzed. As shown in Fig. 2a, RA exhibits a distinct absorption peak at 452 nm and a strong fluorescence emission peak near 580 nm (*λ*_ex_ = 452 nm). Furthermore, the fluorescence emission spectrum of RA partially overlaps with the visible absorption spectrum of N719 (470–700 nm), indicating that the fluorescence emitted by RA can likely be absorbed by N719, providing reliable evidence for FRET.^30^ Next, the AIE characteristics of RA were investigated. As shown in Fig. 2b and c, which displays the fluorescence spectra and relative fluorescence intensity of RA in DMSO/toluene mixtures with varying toluene fractions, the fluorescence intensity increases with the rising content of the poor solvent toluene—a typical AIE behavior. As the toluene proportion increases, the fluorescence intensity of RA enhances significantly due to the aggregation-induced emission effect, reaching a maximum at 80%. This phenomenon is attributed to restricted intramolecular motion promoted by molecular aggregation, which enhances radiative transition. However, when the toluene fraction increases to 90%, fluorescence intensity decreases, accompanied by a blue shift, likely due to overly compact molecular packing causing aggregation-caused quenching, along with molecular orbital energy level reorganization leading to the spectral shift. Finally, the stability of RA and N719 was tested. As shown in Fig. 2d, ethanol solutions of both dyes were irradiated with a 500 W xenon lamp, and absorbance was measured at 10-minutes intervals. Results indicate that both RA and N719 exhibit good stability and are not easily decomposed under xenon lamp irradiation.

### 3.2. Discussion on the maximum adsorption capacity of dye-sensitized titanium dioxide

**Fig. 3:**
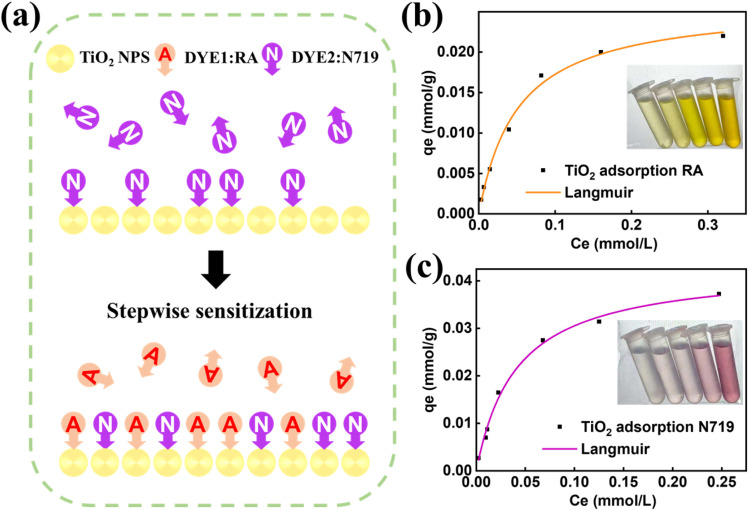
(a) Stepwise cosensitization of TiO_2_ substrates; Langmuir isotherms for the adsorption of (b) RA and (c) N719 dyes by TiO_2_ NPS.

This study employed a stepwise method to sensitize TiO_2_ nanoparticles, and the schematic illustration of this co-sensitization process is presented in Fig. 3a. As depicted in Fig. 3a, the purple “N” symbols represent N719 dye molecules, which first anchor to the high-energy adsorption sites on the TiO_2_ nanoparticle surface (top panel).^31^ The directional arrows indicate that N719, with its robust carboxylate anchoring groups, preferentially occupies these sites to form a uniform initial sensitization layer. Subsequently, the orange “A” symbols (representing RA dye) are introduced for secondary sensitization (bottom panel). The staggered arrangement of “N” and “A” in the final layer visually confirms that the stepwise approach effectively avoids competitive aggregation between the two dyes, enabling RA to fill the remaining low-energy sites and form a dense, homogeneous co-sensitization layer.^32^

To quantitatively evaluate the adsorption behavior of RA and N719 on TiO_2_ NPS, adsorption isotherm experiments were conducted, and the data were fitted using the Langmuir model. The linear form of the Langmuir model is expressed as:

3 *C*_e_/*q*_e_ = 1/*K*_L_*q*_max_ + *C*_e_/*q*_max_

where *C*_e_ (mmol L^−1^) is the equilibrium dye concentration, *q*_e_ (mmol g^−1^) is the equilibrium adsorption amount per unit mass of adsorbent, *q*_max_ (mmol g^−1^) is the maximum adsorption capacity corresponding to monolayer coverage, and *K*_L_ (L mmol^−1^) is the Langmuir equilibrium constant related to adsorption affinity.^33^

Fig. 3b and c show the Langmuir isotherms for RA and N719 adsorption, respectively. The black dots represent experimental data points, while the solid lines denote Langmuir model fits. It is evident that the experimental data closely follow the fitted curves, with high correlation coefficients (*R*^2^ = 0.9939 for RA and *R*^2^ = 0.9955 for N719, as summarized in Table 1). These high *R*^2^ values confirm that the adsorption of both dyes on TiO_2_ NPS conforms well to the Langmuir model, indicating a monolayer adsorption process on a homogeneous surface.

**Table 1:** The isothermal constants of TiO_2_ NPS adsorbing RA and N719 dyes

Model	Langmuir		
Contants	*q*_ma*x*_ (mmol g^−1^)	*K*_L_ (L mmol^−1^)	*R* ^2^
RA	0.02604	19.66	0.9939
N719	0.043	24.51	0.9955

From the slope and intercept of the linear fits, the qmax and *K*_L_ values were derived; see Table 1 for details. The *q*_max_ of N719 (0.043 mmol g^−1^) is approximately 1.65 times higher than that of RA (0.02604 mmol g^−1^), which correlates with our earlier observation that N719 preferentially occupies high-energy adsorption sites. Additionally, the higher *K*_L_ value of N719 (24.51 L mmol^−1^) compared to RA (19.66 L mmol^−1^) indicates a stronger adsorption affinity between N719 and TiO_2_ NPS. These results collectively validate the rationality of the stepwise sensitization strategy: N719 forms a stable initial layer, and RA efficiently fills the remaining sites to achieve broad-spectrum light absorption. The detailed morphology of the co-sensitized TiO_2_ is provided in Fig. S4.

### 3.3. Optimization of PMMA fibers spinning parameters

**Fig. 4:**
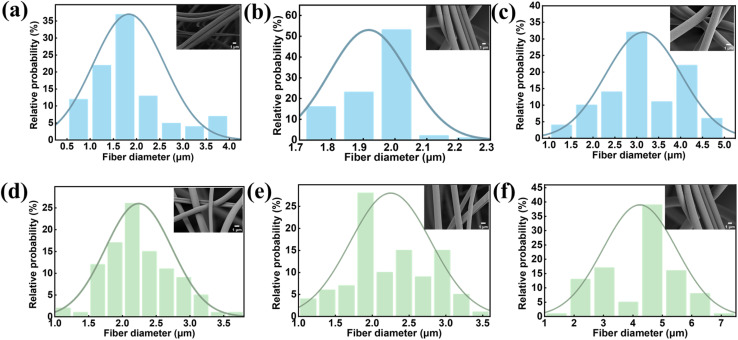
(a–c) The fiber diameter of PMMA fibers obtained at 12 000 rpm with different concentrations of PMMA (23 wt%, 24 wt%, and 25 wt%); (d–f) the fiber diameter of PMMA fibers obtained at 24 wt% with different rotational speeds (8000 rpm, 10 000 rpm, and 12 000 rpm). Insert: SEM images, Scal bar: 1 µm (a–f), 1 µm.

In centrifugal spinning, the solution concentration and spinning speed are critical parameters influencing fiber structure. To investigate the effect of PMMA concentration on fiber morphology, spinning solutions with concentrations ranging from 23 to 25 wt% were electrospun at a fixed speed of 12 000 rpm. As shown in Fig. 4a–c, the fiber diameter increased with rising solution concentration. However, at 25 wt%, the increased viscosity of the spinning solution hindered sufficient solvent evaporation, resulting in the formation of fibers with larger diameters. After optimizing the solution concentration, the influence of centrifugal speed on PMMA fiber formation was further examined. A series of rotational speeds from 8000 to 12 000 rpm was applied at a fixed concentration of 24 wt%. As depicted in Fig. 4d–f, higher rotational speeds enhanced centrifugal force and promoted jet stretching. This not only reduced inter-fiber adhesion but also minimized the risk of fiber breakage, leading to the production of finer fibers. However, when the speed was further increased to 14 000 rpm, the risk of fiber breakage rose significantly, making fiber collection impractical and unsuitable for subsequent applications.^34^

Although the spinneret orifice diameter had a limited impact on fiber morphology, its influence was carefully evaluated. A smaller orifice diameter of 0.3 mm helped maintain solution viscosity and contributed to the formation of thinner fibers during spinning,see in Fig. S5. Through systematic optimization, the following parameters were identified as optimal for PMMA fiber spinning: solution concentration of 24 wt%, spinning speed of 12 000 rpm, and spinneret orifice diameter of 0.3 mm.

### 3.4. Characterization of nanocomposite fibers

#### 3.4.1. Elemental analysis

**Fig. 5:**
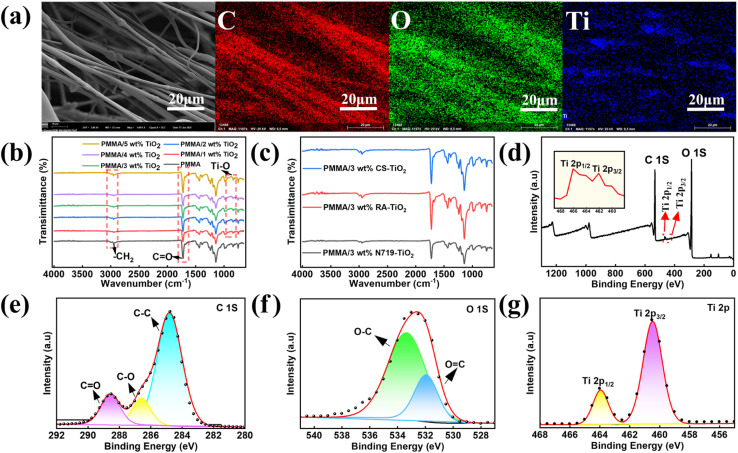
(a) EDS elemental analysis diagrams (C, O, Ti) of PMMA/CS-TiO_2_ membranes; (b) FT-IR spectra of PMMA fibers with different TiO_2_ contents; (c) FT-IR spectrum of PMMA fibers with different dyes sensitized TiO_2_ incorporated into it; (d) XPS spectra of PMMA fibers; Deconvolution XPS spectra of PMMA fibers of C 1s (e), O 1s (f), and Ti 2p (g).

Elemental analysis revealed the presence of carbon, titanium, and oxygen on the substrate surface, see in Fig. 5a. Fig. 5b shows the representative FT-IR spectra of PMMA fibers with different TiO_2_ contents added. The characteristic absorption bands of PMMA were detected in all samples. The strong absorption peak that occurred at 1724 cm^−1^ belonged to the stretching vibration of the PMMA ester carbonyl group (C=O), and the characteristic peak at 1145 cm^−1^ corresponded to the stretching vibration of the ether bond (C–O–C).^35^ The absorption double peaks at 1434 cm^−1^ and 1484 cm^−1^ originate from the asymmetric bending vibration of methyl (–CH_3_) in PMMA, while the absorption bands at 2951 cm^−1^ and 2994 cm^−1^ can refer to the stretching vibration mode of methylene (–CH_2_^−^). Notably, the peak intensity at 2994 cm^−1^ shows a regular change with the increase of TiO_2_ content, indicating that there is a specific interaction between TiO_2_ and PMMA molecular chains. As shown in Fig. S6, the key evidence emerged at 692 cm^−1^, where the newly formed characteristic absorption band was clearly attributed to the Ti–O–Ti skeleton vibration, confirming the successful loading and structural integrity of TiO_2_ nanoparticles in the fibers.^36^ Secondly, as shown in Fig. 5c, the FT-IR spectra of PMMA/N719-TiO_2_, PMMA/RA-TiO_2_, and PMMA/CS-TiO_2_ samples exhibited high similarity. This observation is primarily attributed to the strong characteristic absorption peaks of the PMMA matrix in the infrared region, whose signal intensity significantly overshadowed the subtle spectral differences arising from the various sensitizer monolayers on the TiO_2_ surface, thereby largely masking their potential characteristic vibrational peaks.

Fig. 5e–g shows the XPS spectra of Ti 2p, O 1s band, and C 1s band, respectively. The peak of Ti 2p is located at 458.7 eV and 464.5 eV, which can be attributed to Ti 2p_3/2_ and Ti 2p_1/2_ of TiO_2_.^37^ These signals of Ti 2p_1/2_ and Ti 2p_3/2_ correspond to Ti^4+^ in TiO_2_. The O 1s peaks are located at 531.1 eV and 533.7 eV, corresponding to –OH (surface hydroxyl groups) and Ti^4+^–O (O–Ti–O).^38^ The C 1s are located near 285.0 eV, 287.2 eV, and 289.0 eV, respectively, corresponding to the C–C/C–H bonded carbon. The intermediate peak at 287.2 eV corresponds to the C–O-bonded carbon in the ester group. The characteristic peak at 289.0 eV originates from the carbonyl carbon of the ester (O=C–O).

#### 3.4.2. Mechanical property analysis

**Fig. 6:**
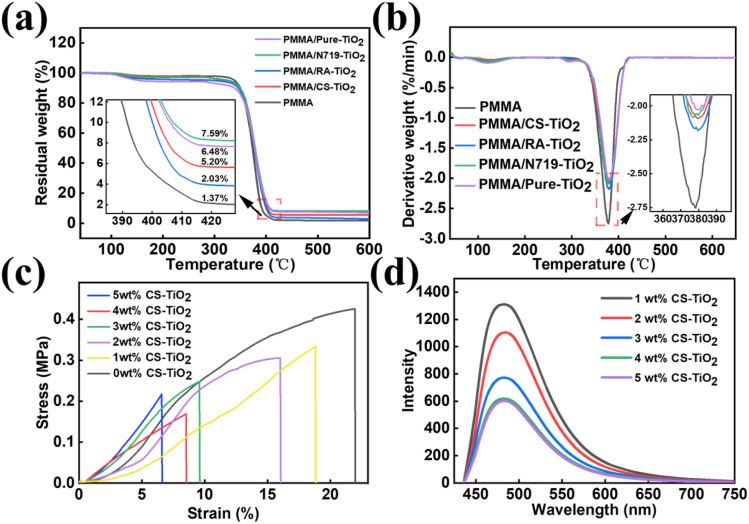
(a and b) The TG-DTG curve of PMMA fibers; (c) stress–strain curves of PMMA fibers with different CS-TiO_2_ concentrations (0–5 wt%); (d) PL spectra of TiO_2_ PMMA fibers with different concentrations (1–5 wt%).

As shown in Fig. 6a and b, the thermal decomposition process of PMMA exhibits typical one-step degradation characteristics: its initial decomposition temperature is 339.2 °C, and the peak temperature corresponding to the maximum decomposition rate is located at 378.5 °C. During this process, the weight loss rate of PMMA reached 98.63%, and the final residual rate was 1.37%, indicating that it was nearly completely pyrolyzed. It is worth noting that the thermal decomposition behaviors of pure TiO_2_ composite micro–nanofibers and different dye-sensitized composite micro–nanofibers show a high degree of consistency with those of pure PMMA fibers. This may be attributed to the uniform dispersion of TiO_2_ nanoparticles and dye molecules in the PMMA matrix, without forming catalytic degradation active sites. The composite process did not disrupt the ester group structure of the PMMA main chain, and its β -fracture mechanism remained stable.^39^

To guarantee the practical operability of the PMMA/CS-TiO_2_ fiber membrane, the mechanical properties of samples with 0–5 wt% PMMA/CS-TiO_2_ loadings were characterized *via* stress–strain tests, with results presented in Fig. 6c. The neat PMMA fiber (0 wt% CS-TiO_2_) shows relatively low break stress, lacking sufficient robustness for cyclic operation. With 1 wt% CS-TiO_2_ introduced, the stress–strain performance is initially enhanced by the reinforcing effect of TiO_2_ nanoparticles. When the loading reaches 3 wt%, the fiber membrane exhibits optimal mechanical properties: it achieves a higher stress level while maintaining moderate ductility, balancing strength and deformability to resist damage during recovery processes. By contrast, further increasing the loading to 4–5 wt% leads to slight performance degradation, likely due to excess CS-TiO_2_ agglomeration weakening interfacial adhesion with the PMMA matrix. Based on this mechanical evaluation, the 3 wt% CS-TiO_2_ loading was selected as the optimal condition for subsequent photocatalytic experiments—ensuring both catalytic activity and practical reusability.^40^

#### 3.4.3. Optical performance analysis

To investigate the photoluminescence (PL) properties of the nanocomposite fibers, PL spectroscopy was performed on PMMA composite micro/nanofibers with different TiO_2_ loadings (1–5 wt%), and the results are presented in Fig. 6d. As clearly shown in Fig. 6d, all samples exhibit a broad PL emission band centered at approximately 500 nm, which originates from the radiative recombination of photoinduced electron–hole pairs (e^−^–h^+^) in the PMMA matrix. The black curve (1 wt% CS-TiO_2_) displays the highest fluorescence intensity, followed by the red curve (2 wt% CS-TiO_2_), while the purple curve (5 wt% CS-TiO_2_) shows the lowest intensity. With the increase of TiO_2_ loading from 1 wt% to 5 wt%, the fluorescence intensity of the composites decreases significantly, indicating an enhanced probability of non-radiative recombination of photogenerated carriers. This is primarily attributed to the introduction of more TiO_2_ nanoparticles, which act as recombination centers and reduce the transfer efficiency of e^−^–h^+^ to the TiO_2_ conduction band.^36^ Consequently, the number of effective charge carriers participating in the generation of reactive oxygen species (*e.g.*, ·OH and ·O_2_^−^) decreases, ultimately leading to a decline in photocatalytic degradation performance.

In addition, fluorescence intensity tests were conducted on PMMA composite micro/nanofibers sensitized with different dyes. As shown in Fig. S7, the PMMA/RA-TiO_2_ sample exhibits the highest fluorescence intensity, PMMA/N719-TiO_2_ the lowest, and PMMA/CS-TiO_2_ an intermediate level. This variation can be explained by the distinct photophysical properties of the dyes: RA, as an organic dye with aggregation-induced emission characteristics, has a molecular structure that favors strong fluorescence emission upon photoexcitation. In contrast, N719, a ruthenium-based metal complex, primarily dissipates excitation energy through non-radiative charge transfer pathways, resulting in inherently weak fluorescence. The intermediate fluorescence intensity of PMMA/CS-TiO_2_ thus reflects the combined effect of RA's strong fluorescence and N719's non-radiative decay, which is consistent with the stepwise co-sensitization design. These PL results further corroborate the charge transfer dynamics in the composite system, providing insights into the structure–activity relationship of the photocatalysts.

### 3.5. Evaluation of photocatalytic performance

Owing to the structural characteristics of the CS-TiO_2_ photocatalyst and the micro–nano fiber membrane, the composite material exhibits high removal efficiency for dye wastewater. Therefore, methylene blue (MB), a typical organic dye, was selected as the target pollutant to investigate the photocatalytic activity with different catalyst loadings.

#### 3.5.1. Effect of catalyst loading

**Fig. 7:**
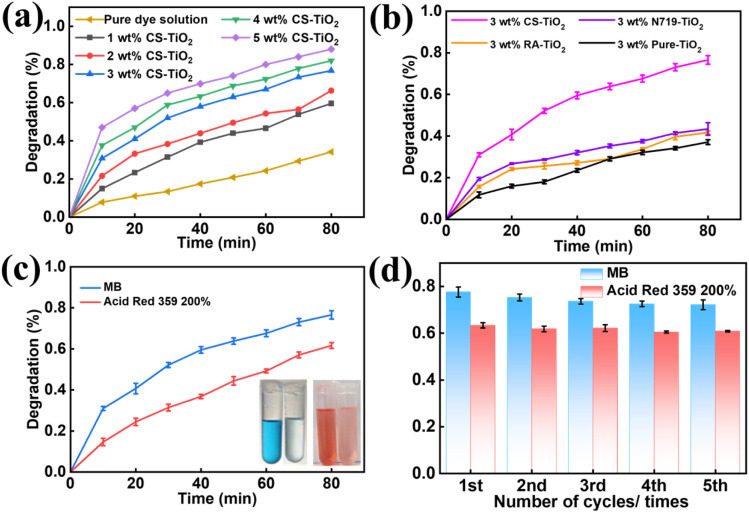
(a) The influence of micro–nano fiber membrane with different CS-TiO_2_ concentrations (1–5 wt%) on the photocatalytic degradation of MB; (b) the influence of micro–nano fiber membrane sensitized with different dyes on the photocatalytic degradation of MB; (c) the efficiency of degradation of MB and acid 3 59 200% by PMMA/3wt% CS-TiO_2_; (d) the recycling performance of PMMA/3wt% CS-TiO_2_.

Degradation studies were conducted with an MB concentration of 10 mg L^−1^ and CS-TiO_2_ concentrations in the micro–nano fiber membrane of 0, 1, 2, 3, 4, and 5 wt%, respectively. Fig. 7a shows the degradation of MB with different CS-TiO_2_ mass concentrations. As illustrated, the degradation of MB by the micro–nano fiber membrane increased with the rising CS-TiO_2_ mass concentration. When the CS-TiO_2_ concentration exceeded 3 wt%, although the surface loading on the membrane increased, the degradation rate did not show a significant enhancement. Further increase in CS-TiO_2_ concentration continued to raise the surface loading, potentially leading to agglomeration of CS-TiO_2_ particles. Consequently, the MB degradation efficiency did not improve markedly. Thus, the optimal CS-TiO_2_ concentration was determined to be 3 wt%.

#### 3.5.2. Effect of different dye sensitizations on TiO_2_Catalytic performance

According to Fig. 7a, the degradation rate of pure dye solution MB is about 30% of the actual concentration, which clearly indicates that MB dye itself has a self-photosensitizer effect. When evaluating the degradation efficiency of other samples, the self-sensitization portion needs to be deducted to accurately reflect the catalytic contribution of the material itself. After calibration based on this, as shown in Fig. 7b, the degradation rate of MB by PMMA/CS-TiO_2_ is about 50%, while the degradation rates of methylene blue wastewater by PMMA/RA-TiO_2_ and PMMA/N719-TiO_2_ are only 10% and 12%, respectively. These results further confirm the complementary spectral effects achieved by the co-sensitization of RA and N719, where N719 mainly covers the visible light wavelength range, while RA effectively supplements the 400 to 500 nanometer wavelength range. The synergistic effect of the two not only significantly expands the range of light response but also greatly enhances the photon capture capability. Similarly, for the acid red 3 59 200% dye wastewater, as shown in Fig. S8, after deducting its 10% self sensitization degradation portion, the net degradation rate of PMMA/CS-TiO_2_ is about 52%.

#### 3.5.3. Application stability of the catalyst

The reusability and stability of a catalyst are pivotal for its cost-effectiveness and practical deployment in wastewater treatment.^41^ As clearly illustrated in Fig. 7d, the 3 wt% PMMA/CS-TiO_2_ fiber membrane exhibited excellent recyclability toward both MB and acid red 3 59 200% over five consecutive photocatalytic cycles under xenon lamp irradiation. After calibrating for self-degradation contributions, PMMA/CS-TiO_2_ exhibited genuine photocatalytic removal efficiencies of approximately 50% for methylene blue and 52% for acid red 3 59 200% in the first cycle. After five cycles, the removal efficiency for methylene blue remained at about 80% of its initial value, corresponding to a net efficiency of approximately 48%, while that for acid red 359 was maintained at about 65% of its initial value, yielding a net efficiency of around 50%. The material demonstrated consistent degradation performance over multiple cycles. The observed slight and gradual decline in activity is a common phenomenon in photocatalytic systems, attributable primarily to minimal catalyst loss during cyclic recovery and minor fouling of the membrane surface by residual dye molecules.

Notably, the PMMA/CS-TiO_2_ fiber membrane retained its structural integrity throughout all cycles, enabling convenient recovery through simple rinsing and filtration instead of cumbersome centrifugation or sedimentation operations. This feature sharply distinguishes it from traditional nano-TiO_2_ powder catalysts, which suffer from severe agglomeration and tedious recovery procedures that easily trigger secondary pollution and elevate operational costs.^42^ In practical wastewater treatment scenarios, this membrane-based design addresses a critical bottleneck: the efficient recovery and reuse of photocatalysts. By eliminating the need for complex separation steps, our strategy significantly reduces operational costs and minimizes environmental risks, thereby enhancing the scalability and applied relevance of the photocatalytic system.^43^ These results not only confirm the satisfactory recyclability and stability of the PMMA/CS-TiO_2_ fiber membrane but also provide a feasible and efficient solution to the long-standing challenge of catalyst recovery in photocatalytic wastewater treatment, highlighting the practical value of this membrane for real-world applications.

### 3.6. Mechanism of photocatalytic dye wastewater degradation by the catalyst

#### 3.6.1. Free radical trapping experiments

To identify the primary active species involved in the photocatalytic degradation of MB, trapping experiments were performed using the 3 wt% CS-TiO_2_ micro–nanofiber membrane. Specific scavengers—*p*-benzoquinone (BQ) for superoxide radicals (·O_2_^−^), ethanol (EtOH) for photogenerated holes (h^+^), and *tert*-butanol (*t*-BuOH) for hydroxyl radicals (·OH)—were introduced into the reaction system under otherwise identical photocatalytic conditions.^44^

**Fig. 8:**
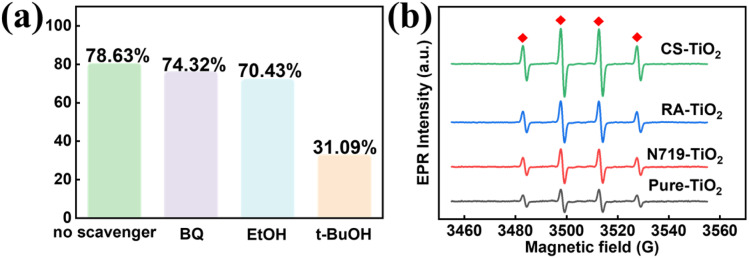
(a) Free radical trapping experiments with 3wt% PMMA/CS-TiO_2_; (b) EPR spectra detecting ·OH radicals generated by PMMA fibers loaded with different dye-sensitized TiO_2_ samples.

As shown in Fig. 8a, the degradation efficiency after 60 min was significantly influenced by the addition of scavengers. The blank group (no scavenger) showed the highest efficiency of 78.63%. The presence of BQ (to trap ·O_2_^−^) resulted in a slight decrease to 74.32%, while EtOH (to trap h^+^) led to a more noticeable reduction to 70.43%. Most strikingly, the system containing *t*-BuOH (to trap ·OH) exhibited a severe suppression of photocatalytic activity, with the efficiency dropping sharply to 31.09%. This represents a reduction of approximately 47.5% compared to the blank group, indicating that the quenching of ·OH had the most detrimental effect on MB degradation.^45^

#### 3.6.2. EPR tests

To directly detect ·OH radicals in the photocatalytic system, Electron Paramagnetic Resonance (EPR) tests with DMPO as the spin trap were performed.^46^ As displayed in Fig. 8b, no signals were observed in the dark, whereas distinct characteristic signals of DMPO-OH adducts appeared for the PMMA/CS-TiO_2_ membrane under xenon lamp irradiation, confirming the photogeneration of ·OH. Furthermore, comparative EPR analysis unveiled the ·OH signal intensity sequence among different samples: CS-TiO_2_ > RA-TiO_2_ > N719-TiO_2_ > Pure-TiO_2_, which is consistent with the photocatalytic performance of these samples obtained from degradation experiments.

#### 3.6.3. Analysis of degradation mechanism

**Fig. 9:**
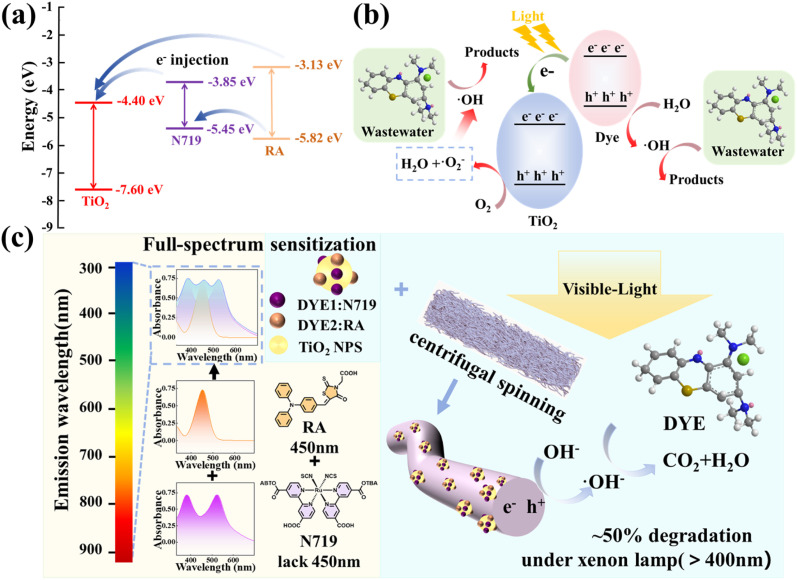
(a) Schematic energy diagram of N719 and RA; (b) the degradation mechanism of pollutants in the PMMA/CS-TiO_2_ visible light catalytic system; (c) schematic diagram of dye co-sensitized TiO_2_ micro–nano fiber membrane and their application in photocatalysis.

The significantly enhanced generation of ·OH radicals in the co-sensitized CS-TiO_2_ sample, as directly evidenced by the EPR intensity trend which followed the order CS-TiO_2_, RA-TiO_2_, N719-TiO_2_, and Pure-TiO_2_ from highest to lowest, is proposed to originate from a synergistic charge transfer mechanism enabled by the complementary energy level structures of N719 and RA. In dye-sensitized systems, efficient electron injection requires the sensitizer's LUMO level to be higher than the conduction band of TiO_2_, positioned at approximately −4.40 eV. Gaussian 16W calculations confirm that the LUMO level of the self-synthesized RA dye meets this prerequisite for sensitizing TiO_2_, as shown in Fig. 9.

As illustrated in Fig. 9b, a complementary charge transfer pathway is inferred. Under visible light, both N719 and RA inject photogenerated electrons into the TiO_2_ CB. Crucially, the matched energy levels facilitate the transfer of photogenerated holes from N719 to RA. This hole transfer concentrates positive charges on RA, which can more effectively oxidize H_2_O or OH^−^ to yield the primary oxidizing agent ·OH. Concurrently, electrons in the TiO_2_ CB are consumed by O_2_, initiating a reduction pathway that may ultimately also contribute to ·OH formation. The specific reaction steps are proposed as follows:^47,48^

4 N719 + *hν* → h^+^N719 + e^−^N719 → N719^+^ + e^−^TiO_2_

5 RA + *hν* → h^+^RA + e^−^RA → RA + e^−^TiO_2_

6 e^−^TiO_2_ + O_2_ → ·O_2_^−^

7 ·O_2_^−^ + 2H^+^ + e^−^TiO_2_ → H_2_O_2_ + e^−^TiO_2_ → ·OH + OH^−^

8 N719^+^ + RA → N719 + RA^+^

9 RA^+^ + H_2_O → RA + ·OH + H^+^

10 ·OH + MB → H_2_O + CO_2_ + products

It should be noted that the above charge transfer behavior is a mechanism inference based on energy level calculations, photocatalytic performance, and free radical experimental results. The improvement of photocatalytic activity is related to more effective charge separation, which is supported by PL quenching, free radical trapping experiments, EPR results, and DFT calculations. In summary, the synergistic effect of these two dyes maximally generates the decisive ·OH, and ultimately achieves efficient photocatalytic degradation of MB through either direct reduction of pollutants or rapid conversion pathways. In summary, as shown in Fig. 9c, N719 and RA have complementary visible light absorption ranges and can produce active oxidants to degrade pollutants.

## 4. Conclusion

In summary, to address the inherent drawbacks of powdered nano-TiO_2_ photocatalysts—including agglomeration, difficult recovery, and low reusability—this study developed a facile centrifugal spinning strategy to fabricate structured PMMA/CS-TiO_2_ micro–nano fiber membranes. This approach transforms nano-catalysts into an easily handled and recyclable macroscopic form, effectively bridging the gap between high catalytic activity and practical operability. The optimized membrane (3 wt% loading) not only exhibited extended visible-light absorption and superior photocatalytic activity, aided by co-sensitization, but also maintained robust mechanical and thermal stability. Most importantly, its excellent recyclability, retaining ∼50% efficiency over five cycles, underscores strong potential for sustainable and cost-effective application in continuous-flow or batch dye wastewater treatment systems.

## Author contributions

Hongyang Cen: writing, conceptualization, investigation, validation. Wei Zhu: supervision, writing—review & editing. Yongqiang Li: methodology, supervision, review & editing, project administration. Yajing Song: investigation, validation. Zhenxin Xu: investigation, validation. Pengjiang Tan: validation. Shuo Cao: investigation. Yonglei Gao: validation. Yi Huang: conceptualization, resources.

## Conflicts of interest

The authors declare that they have no known competing financial interests or personal relationships that could have appeared to influence the work reported in this paper.

## Supplementary Material

RA-016-D5RA09829G-s001

## Data Availability

The data supporting the findings of this study can be obtained from the corresponding author upon request. Due to privacy concerns and other restrictions, the data are not publicly accessible. Supplementary information: the synthesis route and NMR characterization data of the AIE molecule; SEM images and diameter analysis of the PMMA fibers; FT-IR spectra of TiO_2_; fluorescence spectra of sensitized fibers; and photocatalytic self-degradation efficiency data. See DOI: https://doi.org/10.1039/d5ra09829g.
