# Supplementary material for: Recyclable dye-sensitized TiO2 composite membranes with interfacial spectral complementarity for sustainable white-light-driven dye degradation
Source: RSC Adv. 2026 Feb 5;16(8):7590–602. doi: 10.1039/d5ra09829g (PMC12875207; doi:10.1039/d5ra09829g)
Supplement: RA-016-D5RA09829G-s001 [file RA-016-D5RA09829G-s001.pdf]

Supplementary Information (SI) for RSC Advances.
This journal is © The Royal Society of Chemistry 2026

SUPPORTING INFORMATION

Recyclable Dye-Sensitized TiO_2 Composite Membranes with Interfacial Spectral Complementarity for Sustainable White-Light-Driven Dye Degradation

Hongyang Cen[abd1], Wei Zhu[abc1], Yongqiang Li[abcd*], Yajing Song[abd], Zhenxin Xu[abd], Pengjiang Tan[ab], Shuo Cao[abd], Yonglei Gao[ab], Yi Huang[abc]

a State Key Laboratory of Bio-based Fiber Materials, Zhejiang Sci-Tech University, Hangzhou 310018, China
b Engineering Research Center for Eco-Dyeing and Finishing of Textiles, Ministry of Education, Zhejiang Sci-Tech University, Hangzhou 310018, China
c Key Laboratory of Intelligent Textile and Flexible Interconnection of Zhejiang Province, Hangzhou 310018, China
d Tongxiang Research Institute, Zhejiang Sci-Tech University, Tongxiang 314500, China
Corresponding author:
E-mail address: yqqli@163.com (Y. Li).

Table of contents

Fig. S1. Synthesis of AIE **1**. Reagents and conditions: a) NH_4OAc, ACOH, 100 °C, 24 h, 82%.
3
Fig. S2. The ^{1}H-NMR spectrum of RA. 3
Fig. S3. The ^{13}C-NMR spectrum of RA. 4
Fig. S4. Physical images of single-sensitized and co-sensitized TiO_2。 4
Fig.S5. The fiber diameter of PMMA fibers obtained at 24 wt% and 12000 rpm with different Spinning plate aperture (0.3mm, 0.4mm, 0.5mm). Insert: SEM images, Scal bar: 1 μm (a-f), 1 μm.
5
Fig.S6. FT-IR spectra of TiO_2. 5
Fig.S7. FL spectra of PMMA fibers sensitized by different dyes with TiO_2. 6
Fig.S8. Self degradation efficiency of Acid Red 359 200% pure dye solution under xenon lamp irradiation 6

Synthetic procedures of RA

RA: To a stirred solution of 4-formyltriphenylamine (1 eq., 1.10 mmol, 300 mg) and rhodanine 3-acetic acid (1.2 eq., 1.32 mmol, 252 mg) in glacial acetic acid, ammonium acetate (3 eq., 3.30mmol, 254 mg) was added. The solution was heated to reflux for 24 h. After cooling to room temperature, the mixture was filtered and the residue was washed by ethyl acetate/petroleum ether (v/v 1:3) 3×10 mL, pure water 3×10 mL, and absolute ethyl alcohol 3×10 mL. The residue was dried in vacuum to give RA (402 mg, 82%). ^{1}H NMR (400 MHz, $CDCl_3$) δ 7.70 (s, 1H), 7.34 (t, *J* = 7.9 Hz, 6H), 7.26 (s, 1H), 7.16 (dd, *J* = 7.8, 5.3 Hz, 6H), 7.02 (d, *J* = 8.6 Hz, 2H), 4.92 (s, 2H). HRMS(ESI): m/z $[M+1]^+$ calcd for $C_{24}H_{18}N_2O_3S_2$: 447.5390； found 447.0824.

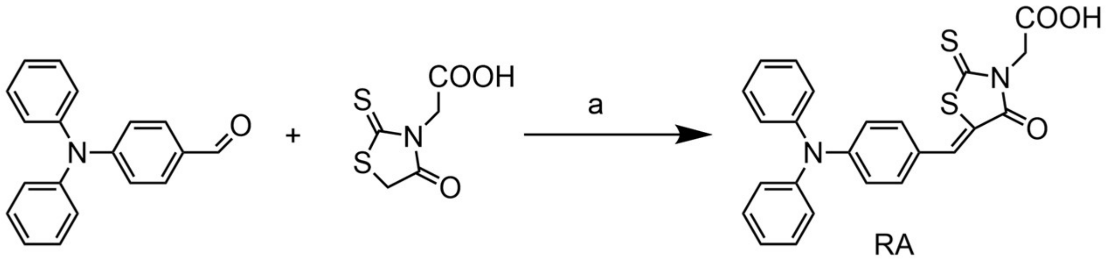

Fig. S1. Synthesis of AIE **1**. Reagents and conditions: a) NH_4OAc, ACOH, 100 °C, 24 h, 82%.

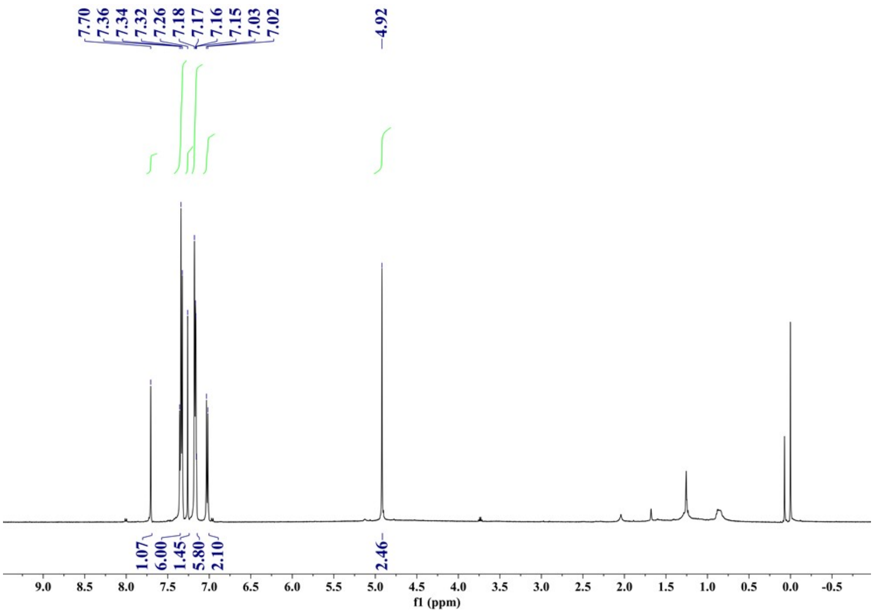

Fig. S2. The ^{1}H-NMR spectrum of RA.

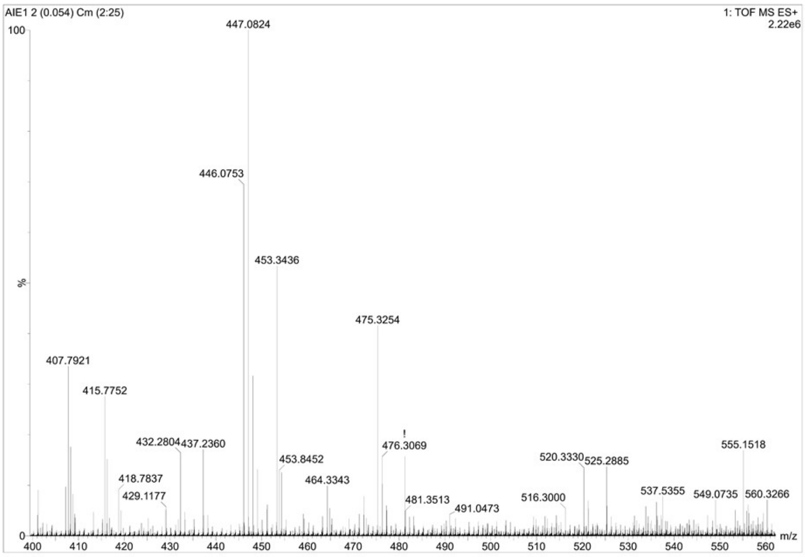

Fig. S3. The ^{13}C-NMR spectrum of RA.

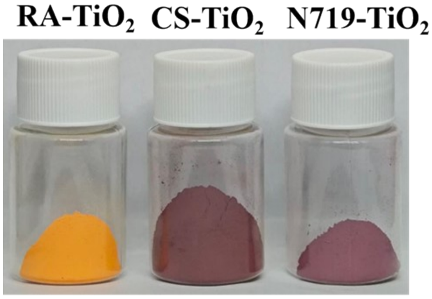

Fig. S4. Physical images of single-sensitized and co-sensitized TiO_2。

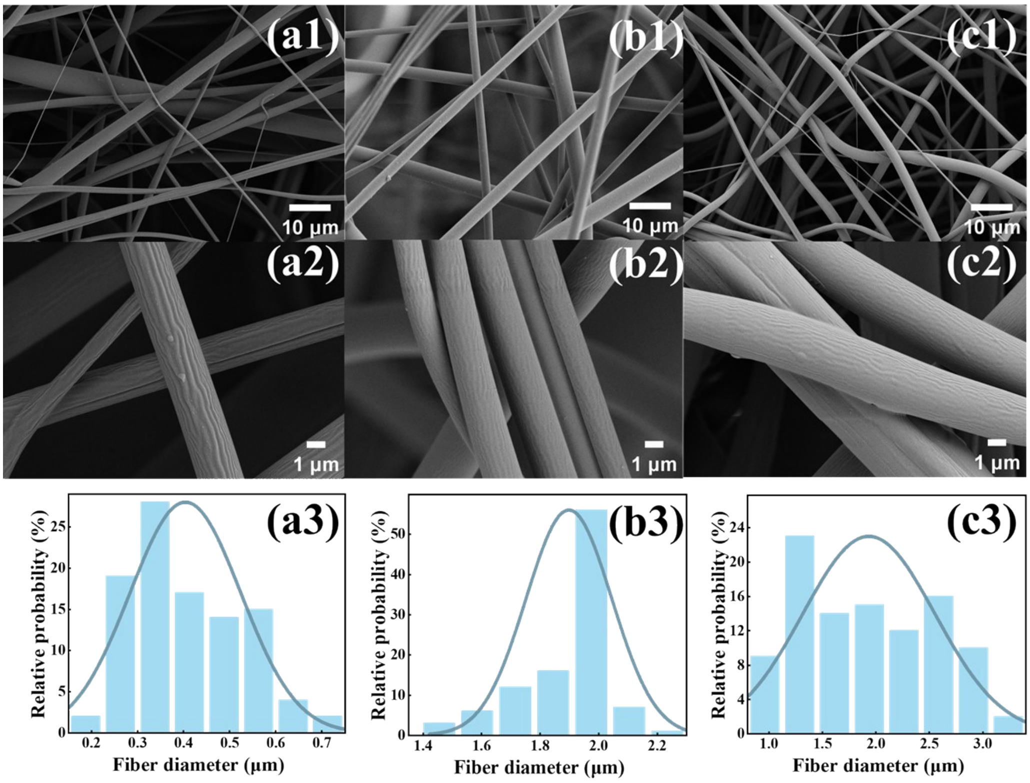

Fig.S5. The fiber diameter of PMMA fibers obtained at 24 wt% and 12000 rpm with different Spinning plate aperture (0.3mm, 0.4mm, 0.5mm). Insert: SEM images, Scal bar: 1 μm (a-f), 1 μm.

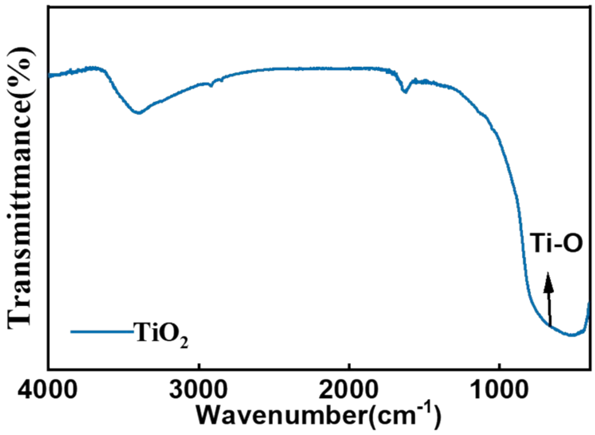

Fig.S6. FT-IR spectra of TiO_2.

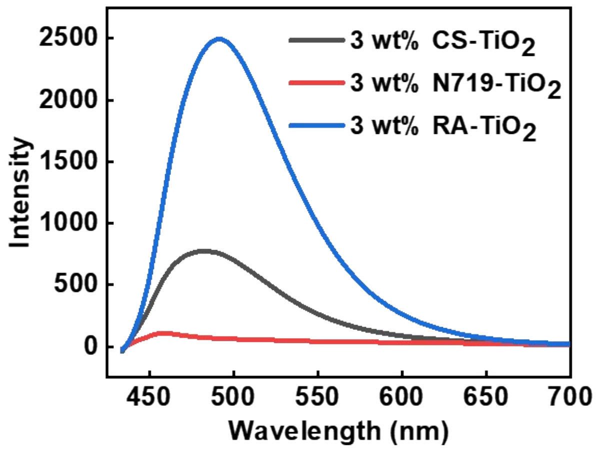

Fig.S7. FL spectra of PMMA fibers sensitized by different dyes with TiO_2.

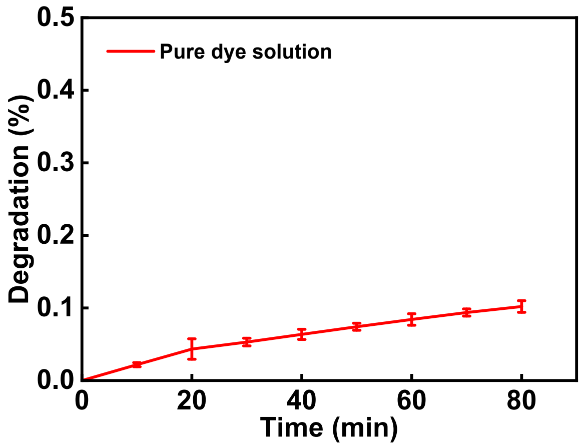

Fig.S8. Self degradation efficiency of Acid Red 359 200% pure dye solution under xenon lamp irradiation
